# Heterosubtypic protective immunity against widely divergent influenza subtypes induced by fusion protein 4sM2 in BALB/c mice

**DOI:** 10.1186/1743-422X-11-21

**Published:** 2014-02-06

**Authors:** Mohammed YE Chowdhury, Soo-Kyung Seo, Ho-Jin Moon, Melbourne R Talactac, Jae-Hoon Kim, Min-Eun Park, Hwa-Young Son, Jong-Soo Lee, Chul-Joong Kim

**Affiliations:** 1 College of Veterinary Medicine (BK21 Plus Program), Chungnam National University, Daejeon 305-764, Republic of Korea; 2 Faculty of Veterinary Medicine, Chittagong Veterinary and Animal Sciences University, Chittagong 4202, Bangladesh; 3 Department of Biomedical Sciences, College of Veterinary Medicine, Iowa State University, Ames, IA, USA

**Keywords:** Broad protection, Humoral immunization, Influenza vaccine, Matrix protein-2

## Abstract

**Background:**

Regular reformulation of currently available vaccines is necessary due to the unpredictable variability of influenza viruses. Therefore, vaccine based on a highly conserved antigen with capability of induction of effective immune responses could be a potential solution. Influenza matrix protein-2 (M2) is highly conserved across influenza subtypes and a promising candidate for a broadly protective influenza vaccine. For the enhancement of broad protection, four tandem copies of consensus M2 gene containing extracellular (ED) and cytoplasmic (CD) without the trans-membrane domain (TM) reconstituted from H1N1, H5N1 and H9N2 influenza viruses were linked and named as 4sM2. The construct was effectively expressed in *Escherichia coli*, purified and proteins were used to immunize BALB/c mice. Humoral and cell-mediated immune responses were investigated following administration.

**Results:**

Mice were intramuscularly immunized with 4sM2 protein 2 times at 2 weeks interval. Two weeks after the last immunization, first humoral and cell mediated immune response specific to sM2 protein were evaluated and the mice were challenged with a lethal dose (10MLD_50_) of divergent subtypes A/EM/Korea/W149/06(H5N1), A/PR/8/34(H1N1), A/Aquatic bird/Korea/W81/2005(H5N2), A/Aquatic bird/Korea/W44/2005(H7N3), and A/Chicken/Korea/116/2004(H9N2) viruses. The efficacy of 4sM2 was evaluated by determining survival rates, body weights and residual lung viral titers. Our studies demonstrate that the survival of mice immunized with 4sM2 was significantly higher (80–100% survival) than that of unimmunized mice (0% survival). We also examined the long lasting protection against heterosubtype H5N2 virus and found that mice vaccinated with 4sM2 displayed 80% of protection even after 6 months of final vaccination.

**Conclusion:**

Taken together, these results suggest that prokaryotic expressed multimeric sM2 protein achieved cross protection against lethal infection of divergent influenza subtypes which are lasting for the long time.

## Background

Influenza A virus, one of the most important pathogen, causes perennial epidemics and occasional pandemics with a huge impact on global health and economy. As a zoonotic agent it has potential to cause diseases not only to the poultry industry, but also to the humans and many species of mammals [1]. Among the two strategies; prevention and therapeutic, prevention being the preferred option to combat the influenza infection [2]. Thus, several kinds of influenza vaccines are developed, such as inactivated whole virus vaccines, split vaccines, subunit vaccines, and virus-like particles [3-6]. Among them, inactivated influenza virus vaccines are the most commonly use to prevent influenza-associated illness [7]. Current inactivated vaccines are formulated as a trivalent blend based on known protective surface antigen hemagglutinin (HA) and neuraminidase (NA) and are designed by predicting the forth coming virus strains, which requires reformulation regularly. However, production of an egg-based trivalent vaccine is time consuming and limits the chance to change the antigen in an emergency situation [8]. Moreover, current strategies using inactivated whole virus vaccines face an annual problem of HA and NA antigenic mismatch with circulating influenza viruses due to repeated antigenic drifting [9]. Therefore, vaccination strategies for broad protectivity against unpredictable influenza viruses need to be developed.

Several approaches are being investigated to develop broadly protective vaccines and focus mainly on the conserved region of the viral matrix protein-2 (M2) and HA proteins of influenza A virus [10]. Compared with HA, M2 is highly conserved among and within different subtypes (Table 1), and is therefore an attractive target for developing a broadly protective vaccine. In particular, the extracellular domain of M2 is considered an appropriate target for a broad spectrum influenza vaccine [11]. The efficacies of different forms of M2-based vaccines have been studied and found that mice immunization with M2 can protect against influenza virus lethal infection [12-14]. However, the protection level was not so significant due to small size and poor immunogenicity of the M2e peptide. Therefore, focus has shifted to the M2 fusion construct using a variety of carrier molecules like M2 peptide-carrier conjugates, baculovirus-expressed M2, M2 fusion proteins, multiple antigenic peptides, and M2 DNA vaccine [14-18]. It has been reported that multimeric form of the M2 fusion protein, such as TLR5 ligand flagellin fused to four tandem copies of M2 induced antibody can protects a lethal challenge of influenza virus in BALB/c mice [13]. Subsequently, these studies have shown that immunization with M2 or a multimeric form of M2 based vaccine with or without a carrier can protect homologous or heterosubtypic influenza virus infections. However, the longevity and breadth of cross protectivities of M2 are not well studied.

**Table 1:** Comparison of sM2 sequence among vaccine and challenge strains

**Virus strain**	**Subtype**	**Amino acid sequence**	**Access no.**
Consensus		MSLLTEVETPTRNGWECKCSDSSEPDRLFFKCIYRRLKYGLKRGPSTEGV	
A/EM/Korea/W149/06	H5N1	MSLLTEVETPTRN **E** WECRCSDSS **D** PDRLFFKCIYRRLKYGLKRGPSTEGV	ABW73743.1
A/Aquaticbird/Korea/W81/2005	H5N2	MSLLTEVETPTRNGWECKCSDSS **D** PDRLFFKCIYRRLKYGLKRGPSTEGV	EU819138.1
A/Puerto Rico/8/34	H1N1	MSLLTEVETPIRN **E** W **G** C **R** C **NG** SS **D** PDRLFFKCIYRR **F** KYGLK **G** GPSTEGV	NC_002016.1
A/Aquaticbird/Korea/W44/2005	H7N3	MSLLTEVETPTRNGWEC **R** CSDSS **D** PDRLFFKCIYRRLKYGLKRGPSTEGV	JN244137.1
A/Chicken/Korea/116/2004	H9N2	MSLLTEVETPTRNGWECKCSDSS **D** P **L** RLFFKCIYRRLKYGLKRGPSTEG **M**	EU819104.1

The amino acids differ from consensus sequence are in bold and underlined.

Therefore, in this study, a construct named 4sM2 using four tandem copies of consensus sM2 gene derived from the analysis of sequences of H1N1, H5N1 and H9N2 influenza viruses without its trans-membrane domain for the induction of broad protection against divergent influenza virus subtypes were developed. The construct was expressed in *E. coli* and potency of the produced immunogen was evaluated in mouse model against lethal doses of mouse adapted influenza A viruses. In addition, the longevity of the immune responses and breadth of cross protection were examined.

## Results

### Confirmation of target protein 4sM2

**Figure 1:**
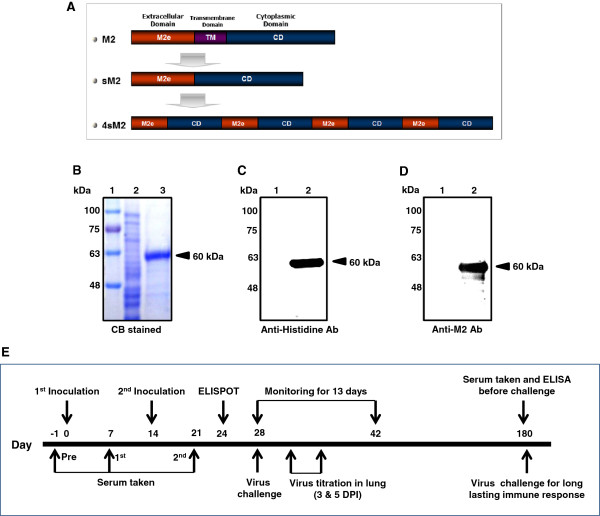
**Construction of plasmid analysis of recombinant proteins and mouse vaccination schedule. (A)** The consensus sM2 genes were cloned into pRSET A vector. **(B)** Purified 4sM2 protein from a prokaryotic expression system was confirmed by Coomassie Brilliant Blue staining and was detected at a molecular weight of 60 kDa. 4sM2 protein expression was verified by Western blot analysis using **(C)** anti 6 × His antibodies and **(D)** antiM2 antibodies. **(E)** Mice were grouped as shown in Table 2 and all groups were intramuscularly administered twice every other week. Sera were collected before the first administration and 1 week after each vaccination. Spleens were excised from three mice in each group of one set 10 days after last immunization. All mice were challenged with a lethal dose of HPAI viruses intranasally and monitored for 13 days at 2 or 24 weeks after the last immunization. At 3 and 5 dpi, lungs were excised from three mice in each group to check the virus titer. Mice from one set were sacrificed for lung histopathology at 5 dpi. Abbreviations: 4sM2, recombinant multimeric sM2 protein; NC, negative control; M, protein marker; CB, Coomassie Brilliant Blue.

For the expression of target proteins with his-tag fusion at the N-term, monomer or multimer consensus sM2 plasmids (sM2 and 4sM2, respectively) were constructed into pRSET A vector. The recombinant proteins were expressed mainly as inclusion bodies in *E. coli*. Refolded inclusion bodies containing the recombinant proteins were purified by His-tag affinity chromatography and dialyzed using permeable cellulose membrane and confirmed by SDS-PAGE and immunoblotting. As shown in Figure 1B, proteins were electrophoresed on the SDS-polyacrylamide gel and stained with Coomassie brilliant blue. After destaining, proteins were observed at the expected molecular weight of 60 kDa (4sM2, Figure 1B) and 15 kDa (sM2, data not shown). Additionally, reactions of 4sM2 protein with mouse anti-Histidin (C-term, Figure 1C) and rabbit anti-M2 antibodies (Figure 1D) were confirmed by immunoblotting. With Coomassie staining and immunoblotting, no other proteins were observed around the expected band which indicate, there were no conformational changes of proteins after refolding from inclusion bodies.

### M2 specific antibody responses to the 4sM2 protein in mice

**Figure 2:**
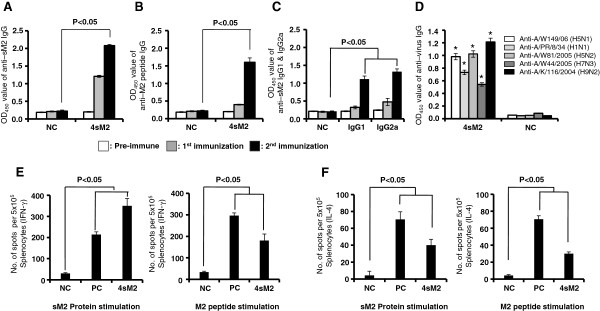
**Detection of sM2-specific humoral and cell mediated immune responses.** Sera were collected at days 0 (pre-immune), 7 (first immunization) and 21 (second immunization) of immunization with the 4sM2 recombinant protein. The absorbance of antibody was detected by indirect ELISA. **(A)** Detection of serum IgG using the sM2 protein as coating antigen. **(B)** Detection of serum IgG using the M2 peptide as coating antigen. **(C)** Detection of serum IgG1 and IgG2a antibody responses in mouse sera. **(D)** Detection of serum IgG using the whole inactivated virus as coating antigen. Splenocytes were harvested 10 days after the last immunization. Cells were re-stimulated *in vitro* with the sM2 protein or M2 peptide and cytokine forming cell spots were determined by ELISPOT assay. IFN-γ and IL-4 spot-forming cells per 5 × 10^5^ splenocytes were determined.** (E)** Splenocytes producing IFN-γ stimulated with the sM2 protein and M2 peptide. **(F)** Splenocytes producing IL-4 stimulated with sM2 protein and M2 peptide. Bars denote mean ± standard deviations. Comparison of groups were analyzed by Student’s t-test and ANOVA; the differences were statistically significant (* *P* < 0.05). Abbreviations: 4sM2, recombinant multimeric sM2 protein; NC, negative control; PC, positive control.

Upon confirmation of protein expression and subsequent purification, groups of mice were immunized intramuscularly (i.m.) with 30 μg of 4sM2 protein at day’s 0 and 14, and sera were collected at day’s -1, 7, and 21 to assess the antibody titer. Serum antibodies were measured by ELISA using the sM2 protein (Figure 2A), M2 peptide (Figure 2B) or inactivated purified virions as a coating antigen. The levels of serum IgG absorbance increased around 10 fold after the second application of 4sM2 protein compared with those observed before immunization (Figure 2A and B). As shown in Figure 2D immune sera collected from mice boosted with 4sM2 showed significant level of antibodies cross reactivities to influenza subtypes H5N1, H5N2 and H9N2 viruses. Importantly 4sM2 immune sera also showed significant levels of cross reactivities to H1N1 and H7N3 subtypes which contains sM2 sequences of 8 and 2 amino acid mismatches respectively (Table 1). Control group of mice that were immunized with 0.85% saline (NC) did not show any responses against protein, peptide or inactivated whole viruses. Therefore this result suggests that 4sM2 is capable to induce antibody which is cross reactive to different subtypes of influenza virus.

M2 specific IgG1 and IgG2a were also tested and found that both IgG1 and IgG2a increased significantly compare to negative control (NC) after the booster immunization (Figure 2C), and IgG2a was predominant than IgG1. These results suggest that the 4sM2 recombinant protein is strongly immunogenic and is also capable of producing both Th1 and Th2 inducing M2 specific IgG1 and IgG2a antibodies.

### 4sM2 induced M2 specific T cell responses

To investigate the broad protective immune mechanism, 4sM2 induced IFN-γ and IL-4 secreting cells in the spleen were determined by ELISPOT. Cells were collected 10 days after the boost immunization and stimulated with sM2 protein or M2 peptide. Significant numbers of IFN-γ secreting cells were observed in the spleen following stimulation with both the sM2 protein (Figure 2E, left) and M2 peptide (Figure 2E, right). We also observed a detectable level of IL-4 secreting splenocytes following stimulation with both the sM2 protein (Figure 2F, left) and the M2 peptide (Figure 2F, right). Mock (un-immunized) mice showed a background level of spot for both the sM2 protein and M2 peptide stimulation. These findings indicate that four tandem copies of consensus sM2 can induce M2-specific IFN-γ and IL-4 secreting T cell responses, which may contribute to the protective immunity.

### Protective efficacy of 4sM2 vaccine against divergent influenza virus subtypes

**Figure 3:**
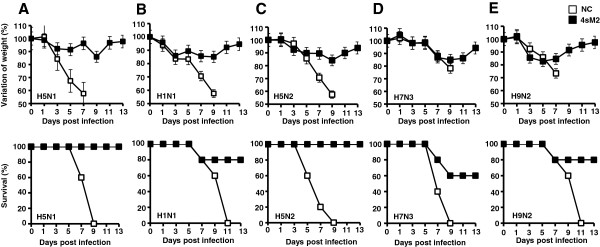
**Protective against divergent influenza virus subtypes.** Five weeks-old female BALB/c mice were immunized twice with 30 μg of 4sM2 protein or 0.85% saline via i.m. Mice were infected intranasally with 10 times the 50% mouse lethal dose (MLD_50_) of mouse-adapted influenza virus subtypes **(A)** A/EM/Korea/W149/06(H5N1), **(B)** A/PR/8/34(H1N1), **(C)** A/Aquatic bird/Korea/W81/2005(H5N2) respectively, **(D)** A/Aquatic bird/Korea/W44/2005(H7N3) and **(E)** A/Chicken/Korea/116/2004(H9N2) at 14 days after the final immunization Variations in body weight from the initial mouse body weight and percent survival were recorded until 13 dpi. The results are expressed as the mean ± standard deviations for the group.

The efficacies of the 4sM2 vaccine against the divergent influenza virus subtypes were investigated. Mice were immunized i.m. twice at 2 weeks interval. Two weeks after the final immunization, mice were challenged intranasally (i.n.) with the 10MLD_50_ of A/EM/Korea/W149/06(H5N1) influenza subtype that contains 2 mismatched amino acids against the sM2 consensus sequence (Table 1). Protective efficacy and morbidity (measured by survival rates and weight losses, respectively) were monitored every other day for 13 days post-infection (dpi); mice were euthanized and considered dead if the original body weight is reduced by >25%. As shown in Figure 3A, immunized mice lost 5–10% of their body weight but conferred 100% survival by 13 dpi after lethal challenge of H5N1 virus (Figure 3A). In contrast, remarkable losses of body weight were observed in unimmunized mice and none of them survived due to lethal infection of H5N1 virus (Figure 3A, bottom).

Next the protection efficiency of 4sM2 vaccine against A/PR/8/34(H1N1) subtype of influenza virus that contains 8 mismatched with sM2 consensus sequence was evaluated. Set of immunized mice were challenged with 10MLD_50_ of the H1N1 virus and protection efficacy were measured as before. Unimmunized mice lost >25% of body weight and all died by 9 dpi whereas mice immunized with 4sM2 showed negligible body weight loss and 80% were survived (Figure 3B). Another set of vaccinated mice were infected with A/Aquatic bird/Korea/W81/2005(H5N2) influenza virus to better understand the degree of cross protection by 4sM2 vaccine. The sM2 sequence of H5N2 contains 1 mismatched against sM2 consensus sequence. All mice in the control group became severely ill (lost weight >25%) and eventually died by 9 dpi. In contrast, the 4sM2 immunized group experienced 19% loss in body weight within 3 to 9 dpi, but started to recover thereafter; 100% of the vaccinated mice were survived the H5N2 virus infection (Figure 3C). The breadths of cross protection of the 4sM2 vaccine against divergent influenza subtypes were further examined. For this, 2 sets of immunized mice were challenged with A/Aquaticbird/Korea/W44/2005(H7N3) or A/Chicken/Korea/116/2004(H9N2) that contain 2 and 3 amino acid mismatched with sM2 consensus sequence respectively. In both cases unimmunized mice were severely ill (lost weight >25%) and died by 9 to 11 dpi. Although, mice immunized with 4sM2 showed little body weight loss, but recovered gradually and finally 60% and 80% survived the H7N3 and H9N2 virus challenges respectively (Figure 3D and E). Taken together, the results showed that immune responses induced by highly conserved 4sM2 vaccine conferred the protection against divergent subtypes of influenza virus lethal infection either it is complete or partial.

### Lung virus titers and histopathology

**Figure 4:**
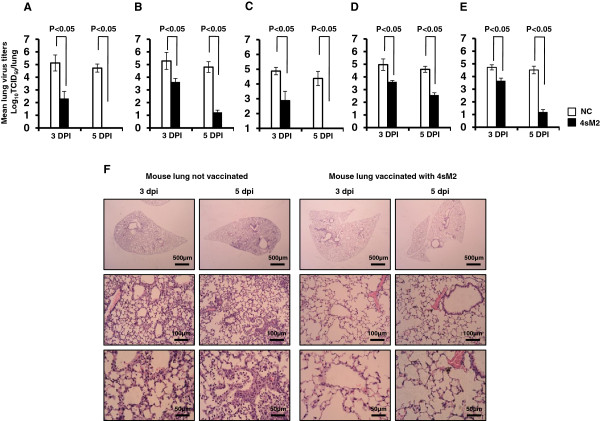
**Lung virus titers and histopathology.** Virus titers in lungs tissues were determined by TCID_50_ in the MDCK cell line at 3 and 5 dpi with **(A)** A/EM/Korea/W149/06(H5N1), **(B)** A/PR/8/34(H1N1), **(C)** A/Aquatic bird/Korea/W81/2005(H5N2) respectively, **(D)** A/Aquaticbird/Korea/W44/2005(H7N3) and **(E)** A/Chicken/Korea/116/2004(H9N2) influenza subtypes. Bars denote mean ± standard deviations. Comparisons of groups were analyzed by Student’s t-test and ANOVA; the differences were statistically significant (*P* < 0.05). **(F)** Lungs of the vaccinated mice show clear alveoli without inflammatory cell infiltration as opposed to the lungs of control mice which revealed the features of severe pneumonitis. Tissues were observed by light microscope with 200× magnification.

Virus titers in the lungs of challenged mice were measured to estimate the virus clearance at 3 and 5 dpi. The 4sM2 immunized mice had significantly reduced lung virus titers at day 3 and had completely cleared the infection by day 5 in case of H5N1 virus challenge (Figure 4A). Similarly, the immunized mice elicited significant reduction of lung virus titers in compare to unimmunized mice by 5 dpi in case of H1N1 and H5N2 influenza virus (Figure 4B and 4C). The clearances of viruses from the lung after challenge with H7N3 (Figure 4D) and H9N2 (Figure 4E) subtypes were also assessed. Both immunized and unimmunized mice showed high lung virus titers at 3 dpi. In contrast, virus titers decreased significantly in immunized group at 5 dpi which correlate the survival result of both H7N3 and H9N2 lethal infection. A histopathological examination was also performed to correlate the virus clearance in the lungs. Representative lungs samples were collected after challenge with H5N2 virus and process to examine under light microscope. As shown in Figure 4F, clear signs of profound pulmonary inflammations were observed in unimmunized mice lung, whereas the mice immunized with 4sM2 showed no significant pulmonary inflammation (Figure 4F). These results demonstrate that consensus 4sM2 protein vaccine induced immune responses strong enough to completely clear the H5N1 and H5N2 subtypes and significantly reduce the virus titers of H1N1, H7N3 and H9N2 influenza subtypes in *vivo*.

### The 4sM2 vaccination induces long lasting cross protection

**Figure 5:**
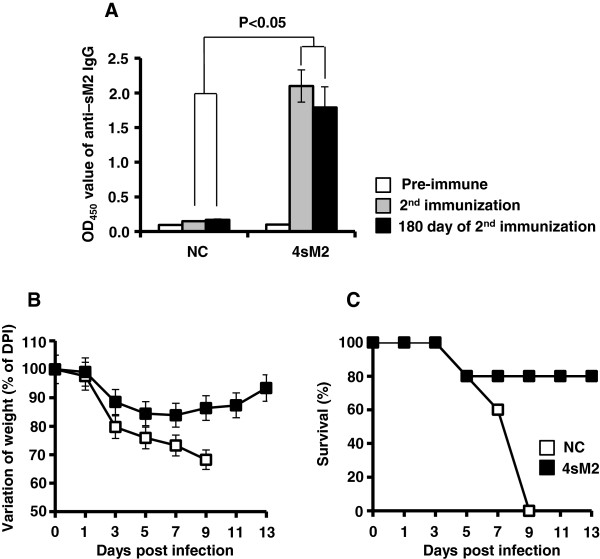
**Long-lasting protections against the heterosubtype influenza virus.** Groups of mice immunized with 30 μg of 4sM2 protein twice at 2 week interval. Control mice were inoculated with 0.85% saline. Sera were collected on days 0, 21, and 180. An ELISA was performed in triplicate using the coated sM2 protein to confirm long lasting antibody level. Mice were challenged with lethal doses of A/Aquaticbird/Korea/W81/2005(H5N2) virus (10× MLD_50_) 6 months after the final immunization. **(A)** Absorbance of the IgG antibody specific to the sM2 protein. **(B)** Percent body weight loss and **(C)** percent survival, after infection with the influenza virus. Bars denote mean ± standard deviations. Comparisons of groups were analyzed by Student’s t-test and ANOVA; the differences were statistically significant (*P* < 0.05). Abbreviations: 4sM2, recombinant multimeric sM2 protein; NC, negative control.

Duration of protection ability is an important criterion for the potential vaccine. For this, the longevity of cross protection by the 4sM2 vaccine was investigated. Mice were immunized according to the schedule mentioned previously, and sera were collected at -1 (pre), 21 (2^nd^), and 180 days after vaccination. Consistent levels of serum IgG specific to sM2 were determined even at 180 days after the final vaccination (Figure 5A). Mice were then challenged with A/Aquatic bird/Korea/W81/2005(H5N2) influenza subtype; morbidity and mortality were checked until 13 dpi. The unimmunized mice showed >25% body weight loss (Figure 5B) and all mice died at 9 dpi whereas the immunized mice survived 80% with negligible body weight loss which was recovered by 13 dpi (Figure 5C). This result demonstrates that 4sM2 vaccine conferred protection even after 6 months of final vaccination against heterosubtype lethal infection.

## Discussion

M2 is one of the most promising conserved antigens, produced by translation from a spliced mRNA derived from influenza gene segment 7, which also cedes for matrix protein M1. It is a type III transmembrane protein in the form of a tetramer that functions as a pH-regulated proton channel and sparsely present on virus particles but is abundant on the surface of virus-infected cells [19]. Various M2e sequences of M2 constructs have been expressed and used as vaccines. In previous study, it has shown that H5 derived vaccines may also protect circulating H1N1 and H3N2 subtypes. Nevertheless, M2 vaccines might also protect against an unexpected subtypes that could cause a pandemic though protection across substantial divergence [16].

Thus, we developed sM2 consensus derived from the analysis of sequences of H5N1, H1N1 and H9N2 subtypes in the database. Considering the previous findings that extracellular domain particularly (aa, 1–13) is highly conserved among the influenza virus subtypes and recognized as epitope for the induction of monoclonal antibodies and protection against influenza virus infection [20-22], sM2 backbone sequence from the H5N1 virus were used. For the possible homology among other subtypes we changed at the position of 14 (E-G), 18 (R-K) and 24 (E-D) and kept unchanged the conserved epitope (aa, 1–13). As shown in sequence alignment, sM2 of consensus sequence has 1–8 mismatches among the subtypes used in this study (Table 1). The developed sM2 plasmids (monomeric and multimeric) were successfully expressed in *E. coli* and subsequently purified on Ni-NTA agarose. The purified proteins were formulated in PBS buffer and tested for its ability to stimulate the immune response and the level of protection against lethal challenge of divergent influenza subtypes.

The M2 specific antibody cannot contribute directly to neutralize virus *in vitro*, the antiviral effect of an M2-based vaccine may be exhibited by antibodies to the M2 antigen through antibody-dependent cell-mediated cytotoxicity [11]. Therefore, induction of the M2 specific antibody level was investigated after two intramuscular doses. High level of M2 specific antibody was identified following immunization with Freund’s adjuvant, which is the most commonly used adjuvant in animal experiment model (Figure 2A and B). Our study also demonstrated that 4sM2 vaccination induces antibodies reacted to purified virions regardless of HA subtypes (Figure 2D).

For investigating the possible mechanism of protection associated with sM2 based vaccine, the isotyping of IgG were performed and found both IgG1 and IgG2a were predominantly induced by 4sM2 vaccinated mice which may contributed to reduction of lung virus titers. Previous study showed that recombinant HA of influenza virus (rHA) with nanoparticles (NP) could induce IgG1 and IgG2a as high as half dose of inactivated virus vaccine (IV) without adjuvant. IgG2a was dominant in case of both rHA and IV when administered with NP adjuvant [23]. On the contrary, Zhao *et al.* demonstrated that M2e peptide with ASP-1 adjuvant could not increase the Th1 (IgG2a) immune response compare to Th2 (IgG1) and suggesting that the selection of an appropriate adjuvant and its unique ability to stimulate functional immune response is critical to the success of M2e based vaccine [24]. However, induction of M2 specific IgG2a antibodies contributes the clearance of viruses [18]. Similarly, reduction of virus titers in the lungs of 4sM2-vaccinated mice after a lethal infection of divergent influenza subtypes (Figure 4A, B, C, D and E) indicated the contribution of 4sM2 induced IgG2a for the clearance of virus. In addition, the 4sM2 vaccination also can reduce the severity of lung damage by inhibiting viral replication and accumulation of inflammatory cells in lung alveolar tissues (Figure 4F).

Since the first report of cross protection by Slepushkin *et al*. [25], numerous studies have done with different approaches to provide cross protection immunity using the conserved M2 sequence. A number of Studies already been conducted focusing on the monomeric M2e proteins expressed in *E. coli*, M2 DNA vaccine, M2 peptide, M2 protein conjugates with different molecules and M2 VLP [14-17]. The routes of administration and the cross protection also been investigated [10,18]. However, for the increasing protection levels, focus goes to multimeric form of M2 protein and peptide. Study showed that 4 × M2e conjugated Mycobacterium tuberculosis HSP70 (mHSP70) fusion protein provided full protection against lethal dose of mouse-adapted H1N1, H3N2, or H9N2 influenza A isolates [26]. A Similar study by Alvarez *et al.*[27] demonstrated that four copies of the M2e peptide to the BLS molecule (*Brucella abortus* derived antigen) were capable to induce 100% protection from viral challenge in BALB/c mice. Recently, Kim *et al.*[28] reported on cross-protection regardless of influenza virus subtypes by tandem repeat of M2e (M2e5×) expressing virus like particles. The multimeric M2e-based vaccines reported have shown to be effective for cross protection when conjugated to other molecules or delivered as a whole virus and mainly based on conserved epitope of M2 ectodomain (M2e) which is small in size and low immunogenic. However, in the present study, we used sM2 constituted both ecto and cytoplasmic domain without the transmembrane domain for the enhancement immunogenicity and breadth of protection, which is utmost important to protect unexpected outbreak of influenza infection.

The cellular immune response plays an important role in vaccination. Previous studies have reported on antibodies and cell-mediated cytotoxicity specific to the M2 antigen and their anti-viral activity [29] and *E. coli* expressed monomeric M2, three copies of M2 fused with ASP-1 significantly induce anti-M2 Th1 and Th2 associated antibodies [24]. Wu *et al*. [30] reported that nucleotide based CpG-ODN adjuvant with M2 peptide significantly increased M2-specific IgG2a and IFN-γ secreting lymphocytes. In agreement with those findings, we examined the Th1-type (IFN-γ) and Th2-type (IL-4) cytokine responses by ELISPOT assay. Heightened levels of IFN-γ were detected in response to stimulation of both the sM2 protein and M2 peptide in mice immunized with the 4sM2 protein but not in non-immunized mice (Figure 2E). Similarly, we observed substantially high levels of IL-4 in immunized mice upon stimulation with the sM2 protein and M2 peptide (Figure 2F). Together, these results indicate that four tandem copies of sM2 with Freud’s adjuvant induced a cellular immune response that may contributed to protecting mice from widely divergent influenza subtypes from both phylogenetic group 1 (H1, H5, H9) and group 2 (H7) [31].

Our study revealed that reconstituted 4sM2 protein which from *E. coli* induced long lasting immunity and conferred protection against a heterosubtype influenza virus lethal infection even at 6 months after final vaccination (Figure 5B and C). Our findings supported by the previous observation that M2 VLP confers long-term immunity and cross protection [18]. Also, a report by Price *et al.* showed long lived NP/M2 specific IgG and IgA antibodies in sera and mucosal sites [32]. In agreement with these findings, we found that the sM2 specific antibody-mediated immunity was long lived (Figure 5A), which is important for any successful vaccine.

## Conclusion

Influenza A viruses are responsible for three major pandemics in the twentieth century and occasionally outbreaks in various hosts such as, humans, avian species, and some types of mammals. It has one of the highest infection rates of all human viruses which can infect people of all ages [33]. Efforts to develop effective influenza vaccines are repeatedly challenged due to the genetic instability of HA and NA [34]. A vaccine consisting of a genetically conserved influenza antigen would provide a second layer of protection against multiple strains and could offer the promise of influenza vaccination in the developing world where the current seasonal strategy is not practical [35]. Therefore, the development of universal influenza vaccines against various subtypes is badly needed and should be studied continuously. In this study, the efficacy of reconstituted multimeric sM2 proteins (4sM2) which expressed in *E. coli* in providing cross-protection against lethal infection of divergent influenza subtypes were demonstrated. We showed evidence that vaccine containing multimeric sM2 which in this case 4sM2 proteins could be potential candidate for inducing cross-protection, as shown against A/EM/Korea/W149/06(H5N1), A/PR/8/34(H1N1), A/Aquatic bird/Korea/W81/2005(H5N2), A/Aquatic bird/Korea/W44/2005(H7N3), and A/Chicken/Korea/116/2004(H9N2) influenza subtypes. The cross reactivity and protective efficacy suggests that 4sM2 protein, could potentially promote protection against influenza subtypes. Overall, our results demonstrate that four tandem copies of consensus sM2 conferred broad protective immune responses against divergent influenza subtypes in a mouse model, suggesting that sM2 could be used to produce a broadly protective influenza vaccine.

## Materials and methods

### Construction of recombinant plasmid with four copies of the sM2 gene

A gene encoding the consensus sM2 containing residues of extracellular and cytoplasmic domain without the transmembrane domain from the analysis of sequences of H5N1, H1N1 and H9N2 subtypes in the database was chemically synthesized (Figure 1A). Plasmid sM2 and 4sM2 were constructed by cloning as described previously [36]. The sM2 gene was modified by adding a *Nhe* I site at the 5′ terminal and *Bam*H I and *Hin*d III sites as well as the termination codons TAA and TGA at the 3′ terminal for cloning into the pRSET A vector. The polymerase chain reaction (PCR) was employed to amplify the gene using the primer pair 5′-CTA GCT AGC ATG TCA TTA TTA ACA-3′ (sense 1), 5′-GAA GAT CTA TGT CAT TAT TAA CA- 3′ (sense 2) and 5′-AAG CTT TTA TCA GGA TCC ACC TGA ACC ACC TGA ACC ACC TGA ACC ACC TTC AAG TTC-3′ (anti sense). Two different primer senses were simultaneously used during this multi-cloning process. The sM2 (sense 1) was ligated with pRSET A using a CoreBio 96 plus thermocycler (CoreBio L&B, Seoul, Korea), whereas sM2 (sense 2) was ligated into the T Easy Vector (Invitrogen, Seoul, Korea). Each plasmid was linearized by RE digestion using *Bam*H I, *Hin*d III for the pRSET A vector and *Bgl* II, *Hin*d III for the T Easy Vector at 37°C for 2 h and purified by phenol/chloroform/ isoamylalcohol treatment. The linearized plasmids were electrophoresed on a 0.9% agarose gel and recovered using a QIAquick Gel Extraction kit (Qiagen, Hilden, Germany) following the manufacturer’s instructions. The pRSET A vector and sM2 insert (sense 2) were ligated with T4 ligase (TaKaRa Bio, Seoul, Korea) at 16°C for 4 h. Sense 1 for sM2 was fused to sense 2 to produce 2sM2. Consequently, 4sM2 was produced by combining 2sM2 (sense 1) to 2sM2 (sense 2). The ligated products were transformed into *E. coli* JM83 competent cells using an electroporation method described previously. The recombinant plasmids were recovered by plasmid DNA extraction following the manufacturer’s instructions using Accuprep Plasmid Mini-prep (Bioneer, Daejeon, Korea). The profiles of the recombinant plasmids were confirmed by restriction endonuclease digestion and DNA sequencing (Solgent, Seoul, Korea).

### Expression of 4sM2 proteins in *E. coli*

Proteins sM2 and 4sM2 were generated using an *E. coli* expression system as described previously [37,38]. Briefly, recombinant plasmids were introduced into the *E. coli* BL21 (DE3) strain using the heat shock method of transformation. A colony was seeded in 5 ml LB broth supplemented with 100 μg/ml ampicillin and 35 μg/ml chloramphenicol and grown at 37°C with shaking. The overnight culture was transferred to 800 ml fresh LB medium and cultured at 37°C with 200 rpm shaking. When the culture reached an optical density (OD) of 600 nm (OD_600_) at 0.6, expression of the target proteins were induced by adding 2 mM isopropyl-β-D-thiogalactopyranoside (99% purity; Bio Basic, Ontario, Canada) and incubating for another 12 h at 30°C. Cultures were then harvested by centrifugation at 6,000 × g for 20 min at 4°C. The cell pellets were stored at -20°C overnight.

### Isolation, solubilization and refolding of protein from inclusion bodies (IBs)

For the isolation of inclusion bodies (IBs), cell pellets were thawed in ice and re-suspended in 20 ml cold buffer containing 20 mM Tris-HCL, 0.5 M NaCl, 10% glycerol and protease inhibitor (1 mM phenyl methyl sulfonyl fluoride, Sigma-Aldrich, Seoul, Korea). Bacterial lyses was performed by sonication for 3 min with an interval of 2 s pulses and 1 s resting at 25% amplitude and centrifuged (12,000 × *g*, 20 min) at 4°C. Supernatant was removed and remaining pellet retained as IBs. The collected pellet was resuspended with denaturing buffer (20 mM Tris-HCl, 0.5 M NaCl, 10% glycerol and 6 M urea) followed by sonication for 1 min and centrifuged as before. Debris supernatant was discarded; remaining pellet was resuspended with denaturing buffer II (20 mM Tris-HCl, 0.5 M NaCl, 8 M urea, pH 8.0) and kept in 4°C with shaking overnight. The pellet was sonicated and centrifuged as before. Supernatant was separated as rescued protein from IBs, which is ready for purification [39].

### Purification, dialysis and confirmation of 4sM2 specific protein

The target proteins were purified by His-tag affinity chromatography (Qiagen, USA), dialyzed using a permeable cellulose membrane (molecular cut-off: 12–14 kDa, Spectrum Laboratories, East Tamaki, Auckland, USA) for 24 h at 4°C. The dialyzed target proteins were quantified using the Bradford assays (Bio-Rad, Hercules, CA, USA) and confirmed by sodium dodecyl sulfate polyacrylamide gel electrophoresis (SDS-PAGE). For immune detection of protein, the membranes were probed with mouse anti-histidine antibodies (1:500, Invitrogen, Carlsbad, CA, USA) and rabbit anti-M2 antibodies (1:1000). Rabbit anti-M2 antibody used in this experiment was generated by i.m. inoculation of KLH conjugated M2 peptide to the rabbit, two times of 2 weeks interval. Membranes were reacted with a 1:1000 dilution of anti-mouse or anti-rabbit IgG HRP. Finally, the target proteins were detected using the WEST-ZOL plus Western Blot Detection System (iNtRON Biotechnology, Gyeonggi-do, South Korea) and visualized by enhanced chemiluminescence (ECL). The purified proteins were used as a vaccine [23].

### Animals, viruses, and vaccination

Preliminary experiment was conducted to determine the doses and efficacy of sM2 and 4sM2 protein on influenza virus infection. Mice vaccinated with sM2 (10 μg and 30 μg) showed 40% and 60% survival respectively while the mice vaccinated with 4sM2 (10 μg) registered 60% survival and the 4sM2 (30 μg) showed 100% survival against lethal infection of H5N1 (data not shown). In this study, monomeric sM2 protein was used as coating antigen for ELISA and stimulator in ELISPOT, multimeric 4sM2 protein used for vaccine study. For this, a total of 138 female BALB/c mice (5 weeks old) were purchased from Samtako (Seoul, Korea) and acclimated for 7 days at room temperature prior to use. Mice were divided into six experimental sets (Table 2). Four sets contained two groups of 11 mice each. One set had two groups of 17 (6 mice for lung histopathology at 3 and 5 dpi) mice each. The remaining sets had two groups containing eight mice each (for the long lasting and CTL response experiment). Mice were vaccinated i.m. with 30 μg of 4sM2 protein or 0.85% saline at 2 weeks interval as illustrated in Figure 1D. Mice inoculated with 0.85% saline considered as a negative control (NC). The first injection included Freund’s complete adjuvant, and 2 weeks later mice were given a boost with 4sM2 or 0.85% saline in Freund’s incomplete adjuvant.

**Table 2:** The design groups for mouse experiment

**No. of Sets**	**Vaccine**	**Adjuvant**	**Route**	**Number of mouse tested for (in each group)**					
				**Total in each group**	**Virus titration** **(3 and 5 dpi)**	**Survival**	**Lung Histopathology** **(3 and 5 dpi)**	**ELISPOT**	**Challenge viruses**
4	0.85% saline	Freund’s	i.m.	11	3 for each time	5	-	-	H5N1 H1N1 H7N3 H9N2
	4sM2	Freund’s		11	3 for each time	5	-	-	
1	0.85% saline	Freund’s		17	3 for each time	5	3 + 3	-	H5N2
	4sM2	Freund’s		17	3 for each time	5	3 + 3	-	
1	0.85% saline	Freund’s		8	-	5 (long lasting)	-	3	H5N2
	4sM2	Freund’s		8	-	5 (long lasting)	-	3	

The highly pathogenic (HPAI) A/EM/Korea/W149/06(H5N1), A/Puerto Rico/8/34(H1N1), A/Aquatic bird /Korea/W81/2005(H5N2), A/Aquatic bird/Korea/W44/2005(H7N3) and A/Chicken/Korea/116/2004(H9N2) influenza subtypes used in this study, were obtained from the virus collection at the College of Medicine and Medical Research Institute, Chungbuk National University, Cheongju, Republic of Korea. All viruses were propagated in allantoic fluid from 10-day-old chicken embryos.

### Sample collection

Blood were collected to analyze serum antibody levels at 0, 7, 21, and 180 days after vaccination. Blood were collected from the retro-orbital plexus, incubated at room temperature for 30 min; sera were separated by centrifugation (12,000 × g, 5 min) and stored at -20°C until analysis. Ether narcosis-anesthetized mice were bled from the heart with a syringe, dissected to expose the thoracic cavity, and the lungs were collected aseptically to determine lung virus titer and lung histopathology. Samples for the lung virus titer were stored at -70°C, and the histopathology samples were fixed in 10% formalin until analysis [36,40].

### Evaluation of antibody

The antibody level specific to sM2 was evaluated by ELISA as described previously [23,41]. Briefly, 96-well immunosorbent plates (Nunc-Immuno Plate MaxiSorp; Nunc Life Technologies, Basel, Switzerland) were sensitized with 3 μg/ml of sM2 protein, M2 peptide or inactivated purified virions for 12 h at 4°C then washed three times with PBS (pH 7.4) containing 0.05% Tween 20 (PBS-T). The wells were blocked with 300 μl of 10% skim milk in PBS for 2 h at room temperature followed by washing again with PBS-T. Sera were diluted 1: 500 for protein and peptide and 1:100 for virus coated plates, added in triplicate, and incubated for 2 h at 37°C. Following another round of washing rabbit anti-mouse IgG HRP antibody (Sigma, Seoul, Korea) was added to each well (1:1000), and incubated for an additional 2 h at 37°C. Substrate solutions containing tetramethylbenzidine and H_2_O_2_ were added after final washing. The reaction performed at room temperature and terminated immediately with stop solution (2 N H_2_SO_4_). Optical density was measured at 450_nm_ using an ELISA auto reader (Molecular Devices, Sunnyvale, CA, USA).

IgG isotyping was performed under the same conditions described for the IgG ELISA except for the secondary antibody. After the primary antibody reaction, the ELISA plates were incubated with 1000-fold diluted rabbit anti-mouse IgG1 HRP and IgG2a HRP (Zymed, San Francisco, CA, USA) for 2 h at 37°C. Subsequent steps were performed as described for the IgG ELISA.

### Enzyme-linked immunosorbant spot (ELISPOT) assay

Cytokine ELISPOTs were developed and counted as described previously to detect and compare the T-cell response to the 4sM2 protein [42,43]. Briefly, BD ELISPOT 96-well plates were coated with anti-mouse interferon IFN-γ or interleukin IL-4 capture antibodies in 100 μl PBS/well and incubated at 4°C overnight. The plates were blocked with complete RPMI 1640 medium containing 10% fetal bovine serum (Invitrogen, Carlsbad, CA, USA), and incubated in RT for 2 h. Freshly isolated splenocytes were added at 5 × 10^4^ cells/well in media containing the sM2 protein (1 μg/well) or M2 peptide (1 μg/well) or only medium (negative control), or 5 μg/ml phytohemagglutinin (positive control, Invitrogen, Carlsbad, CA, USA). Plates were incubated for 48 h at 37°C in 5% CO_2_. After discarding the cells, the plates were treated sequentially with biotinylated anti-mouse IFN-γ and IL-4 antibodies, streptavidin-HRP, and substrate solution. Finally, the plates were washed with deionized water and dried for at least 2 h in the dark. Spots were counted automatically using an Immuno Scan Entry analyzer (Cellular Technology Ltd., Shaker Heights, OH, USA).

### Virus challenge

Mice were anesthetized by ether narcosis and infected intranasally with 10MLD_50_ of challenge viruses in 20 μl PBS. The MLD_50_ of A/EM/Korea/W149/06(H5N1), A/PuertoRico/8/34(H1N1), A/Aquatic bird /Korea/W81/2005(H5N2), A/Aquatic bird/Korea/W44/2005(H7N3), and A/Chicken/Korea/116/2004(H9N2) viruses were determined in 8 week old naive BALB/c mice. Lungs were collected at 3 and 5 dpi to measure lung virus titers and assess lung histopathology. Remaining mice were monitored for body weight changes and survival. Three mice from one set were selected at random 10 days after the final vaccination to analyze the T-cell immune responses. The remaining mice from the same set were challenged at 180 days (after final vaccination) for the long lasting protection assay.

### Virus quantification

Lung virus titers were determined as the 50% tissue culture infectious dose (TCID_50_) as described previously [44,45]. Briefly, lung tissues were homogenized in 500 μl PBS containing antibiotic and antimycotic compounds (Gibco, Grand Island, NY, USA). The supernatants were collected after centrifugation (12,000 × g, 15 min) of mechanically homogenized lung samples. MDCK cells were inoculated with a 10-fold serial dilution of sample and incubated at 37°C in a humid atmosphere of 5% CO_2_ for 1 h. After 1 h of absorption, media was removed and overlay medium containing L-1-tosylamido-2-phenylethyl chloromethyl ketone (TPCK) trypsin (Thermo Fisher Scientific, Rockford, USA) was added to the infected cells and incubated for 3 days. Viral cytopathic effects were observed daily, and titers were determined by the HA test. For HA, chicken red blood cells (0.5%) were added to 50 μl of cell supernatant and incubated for 30 min. The virus titer of each sample was expressed as 50% tissue infected doses using the Reed-Muench method.

### Histopathology

Lungs were collected aseptically at 3 and 5 days post infection. Tissues were fixed in 10% formalin solution, embedded in paraffin, sectioned, and stained with eosin. Tissue sections were examined under a light microscope to assess the pathological changes [46].

### Statistics

Data are presented as means ± standard deviations and are representative of at least three independent experiments. Differences between groups were analyzed by analysis of variance (ANOVA) and means were compared by Student’s t-test. *P*-values less than 0.05 were regarded as significant.

### Ethics statement

The research protocols for the use of mice in this study were conducted following approval from the Institutional Animal Care and Use Committee of Bioleaders Corporation (Reference number BLS-ABSL-10-011). Animal experiments were conducted in bio-safety level BSL-2 and BSL-3^+^ laboratory facilities.

## Competing interests

None of the authors have any financial or personal relationships with other people or organizations that could inappropriately influence or bias this study.

## Authors’ contributions

CJK and JSL was the principle investigator, wrote the grant application and supervised the study. MYEC and SSK performed the experiments and wrote the manuscript. HJM, MRT, JHK and MEP performed cell culture experiments and data analysis. HYS did histopathology. All authors have read and approved of final manuscript.
